# The Impact of Different Inoculation Schemes on the Microbiota, Physicochemical and Sensory Characteristics of Greek Kopanisti Cheese throughout Production and Ripening

**DOI:** 10.3390/microorganisms11010066

**Published:** 2022-12-26

**Authors:** Maria Kazou, Alkmini Gavriil, Olga Kalagkatsi, Theodoros Paschos, Effie Tsakalidou

**Affiliations:** Laboratory of Dairy Research, Department of Food Science and Human Nutrition, Agricultural University of Athens, 11855 Athens, Greece

**Keywords:** Kopanisti cheese, PDO cheese, starters, adjuncts, lactic acid bacteria, yeasts, metataxonomics, microbiota, physicochemical, sensory

## Abstract

Kopanisti is a Greek PDO cheese, which is traditionally produced by the addition of an amount of over-mature Kopanisti, called Mana Kopanisti, to initiate cheese ripening. The aim of this study was the production of four types of Kopanisti cheese (A–D) using pasteurized cow milk, and a combination of the following starters/adjuncts in order to test their ability to be used in Kopanisti cheese production: A: *Lactococcus lactis* subsp. *lactis* and *Lacticaseibacillus paracasei*, B: *L. lactis* and *Lc. paracasei*/Mana Kopanisti, C: *L. lactis* and *Lc. paracasei*/*Ligilactobacillus acidipiscis* and *Loigolactobacillus rennini*, D: *Lig. acidipiscis* and *Loig. rennini*. Throughout production and ripening, classical microbiological, metataxonomics and physicochemical analyses were employed, while the final products (Day 35) were subjected to sensory analysis as well. Most interestingly, beta-diversity analysis of the metataxonomics data revealed the clusters constructed among the Kopanisti types based on the different inoculation schemes. On day 35, Kopanisti A–C types clustered together due to their similar 16S microbiota, while Kopanisti D was highly differentiated. On the contrary, ITS data clustered Kopanisti B and C together, while Kopanisti A and D were grouped seperately. Finally, based on the sensory evaluation, Kopanisti C appeared to have the most suitable bacteria cocktail for the Kopanisti cheese production. Therefore, not only were the conventional starters used, but also the *Lig. acidipiscis* and *Loig. rennini* strains could be used in a standardized Kopanisti cheese production that could lead to final products of high quality and safety.

## 1. Introduction

Kopanisti is a Greek Protected Designation of Origin (PDO) cheese (Official Government Gazette of the Hellenic Parliament 16/14.1.94 and 101/16.2.94), which is exclusively produced in the Cyclades islands. It is a soft cheese, characterized by a smooth texture, good spreadability and a rich, strong and peppery flavour similar to that of blue cheeses [1,2].

Kopanisti is produced from raw or slightly thermized cow, sheep and goat milk or mixtures of them, while recently, the addition of butter up to 15% (*w*/*w*) has been allowed (Official Government Gazette of the Hellenic Parliament 16/14.1.94 and 101/16.2.94). Traditionally, no starter cultures are used and the cheese making relies on the indigenous milk- and cheese-making plant microbiota as well as the addition (up to 10%, *w*/*w*) of the so-called Mana Kopanisti, which is over-mature Kopanisti used as an inoculum for the back-slopping production of Kopanisti [3,4].

So far, Kopanisti has been studied using mainly conventional microbiological and physicochemical approaches [1,2,3,5,6,7,8,9,10]. However, it is well known that culture-dependent methods often fail to detect microorganisms, which are either stressed, damaged or non-culturable in the synthetic microbiological media used [11,12,13,14]. Therefore, classical microbiological analysis cannot be used alone to characterize food microbiota, even when combined with molecular techniques. The limitations of the culture-dependent methods have led to the development of high-throughput sequencing (HTS)-based approaches to study the structure of microbial communities through culture-independent approaches. Among these approaches, metataxonomics is the most widely used for the characterization of food microbiota [15,16,17].

It should be noted though, that culture-dependent methods cannot be completely substituted by the culture-independent ones. Cultivation and isolation of microorganisms remains strictly necessary to discover new species/strains that could be used as starter/adjunct cultures in food fermentations at industrial scale. In this perspective, a few years ago our group [3] analyzed one 3-month-old Kopanisti cheese from Tinos island and one 2-year-old Mana Kopanisti from Mykonos island and isolated only strains of two lactic acid bacteria (LAB) species, namely *Ligilactobacillus acidipiscis* and *Loigolactobacillus rennini*. All isolates were salt tolerant and characterized by their ability to produce a range of cheese aroma-related compounds [3]. Based on the above results, two strains, namely *Lig. acidipiscis* ACA-DC 1533 and *Loig. rennini* ACA-DC 565, were fully sequenced and the genomes were annotated [18,19,20].

Although there are several cheese types produced by raw milk and the cheese-making process is driven by the microbes that naturally occur in milk and the cheese-making environment [21,22], nowadays, the production of most cheeses requires, among others, the use of pasteurized milk and a properly designed cocktail of starter, and sometimes adjunct cultures, depending on the type of cheese. The use of starters/adjuncts results not only in better controlled and more reproducible cheese-making process, but also in final products with standardized quality and safety [23,24].

Since the production of Kopanisti is still very limited and only in small-scale dairy industries, the aim of the present study was the production of Kopanisti cheese using pasteurized cow milk and different bacteria species employing as starter/adjunct cultures that are either commonly used in the production of soft cheeses or have been isolated from artisanal Kopanisti, in order to study their impact on the microbial, physicochemical and sensorial profile throughout cheese production and ripening, and thus, their ability to be used as suitable starter or adjunct cultures in Kopanisti cheese production in large-scale dairy industries.

## 2. Materials and Methods

### 2.1. Cheese Making and Sampling

Four different Kopanisti types (A, B, C and D) were produced using commercial full fat pasteurized cow milk and four different combinations of starter/adjuncts cultures. In addition, Mana Kopanisti was also added in one Kopanisti type, since it is a common practice followed by traditional local producers. The Kopanisti types produced were as follows: (i) Kopanisti A: *L. lactis* ACA-DC 57 (2%, *v*/*v*) and *Lc. paracasei* ACA-DC 116 (1%, *v*/*v*); (ii) Kopanisti B: *L. lactis* ACA-DC 57 (2%, *v*/*v*), *Lc. paracasei* ACA-DC 116 (1%, *v*/*v*) and Mana Kopanisti (1%, *w*/*w*; purchased from Tinos island); (iii) Kopanisti C: *L. lactis* ACA-DC 57 (2%, *v*/*v*), *Lc. paracasei* ACA-DC 116 (1%, *v*/*v*), *Lig. acidipiscis* ACA-DC 1533 (2%, *v*/*v*) and *Loig. rennini* ACA-DC 565 (2%, *v*/*v*); and (iv) Kopanisti D: *Lig. acidipiscis* ACA-DC 1533 (2%, *v*/*v*) and *Loig. rennini* ACA-DC 565 (2%, *v*/*v*). For the preparation of the cheese-making inocula, all strains were cultured in the same milk used for cheese production for 24 h at 30 °C.

Cheese production, performed in triplicates for each cheese type, was as follows. Starter cultures were inoculated in 10 L milk pre-heated at 30 °C and then 1.5 mL CaCl_2_ solution (50%, *v*/*v*, Danisco, Copenhagen, Denmark) was added. After 2 h under periodic stirring, rennet (0.15 g, Chr. Hansen, Hoersholm, Denmark) was added, and milk was left at rest for 2 h at 30 °C for coagulation. The curd was then cut into pieces (1 cm size), left for another 10 min, and then transferred into cheesecloths to drain under pressure (1 kg weight) for 20–24 h at 18 °C. The drained curd was mixed with 2% (*w*/*w*) fine salt; in the case of Kopanisti B, Mana Kopanisti was added just before salting. Finally, salted curds were transferred into glass pots and left for 35 days at 18 °C for ripening.

Cheeses were monitored throughout ripening (days 1, 7, 21 and 35) with respect to their microbiological and physicochemical profiles, while the final products were subjected to sensory analysis.

### 2.2. Microbiological Analysis and Isolation of Colonies

The following groups of microorganisms were enumerated in pasteurized milk, Mana Kopanisti and Kopanisti cheese samples collected during ripening: (i) total mesophilic counts on Milk Plate Count Agar (MPCA; LabM Ltd., Lancashire, UK) at 30 °C for 3 days; (ii) mesophilic lactobacilli on double-layered MRS Agar (Condalab, Madrid, Spain) at 30 °C for 3 days; (iii) thermophilic lactobacilli on double-layered MRS Agar (Condalab) at 42 °C for 3–4 days; (iv) mesophilic cocci on M17 Agar (Condalab) at 30 °C for 3 days; (v) thermophilic cocci on M17 Agar (Condalab) at 42 °C for 3 days; (vi) Non Starter LAB (NSLAB) on double-layered Rogosa agar (Condalab) at 30 °C for 5 days; (vii) enterococci on Kanamycin Aesculin Azide (KAA) Agar (Merck, Darmstadt, Germany) at 37 °C for 24 h; (viii) psychrotrophic bacteria on MPCA (LabM Ltd.) at 4 °C for 10–14 days, (ix) enterobacteria on double-layered Violet Red Bile Glucose Agar (VRBGA; Condalab) at 37 °C for 24 h; and (x) yeasts on Yeast Glucose Chloramphenicol (YGC) Agar (Condalab) at 25 °C for 5 days. Cycloheximide (Merck, Darmstadt, Germany) was added (50 μg mL^−1^) to media (ii–v) to eliminate the growth of yeasts. Results were expressed as log CFU mL^−1^ (milk) or g^−1^ (Mana Kopanisti and Kopanisti cheese samples).

Based on colony morphology, isolates were collected from the enumeration media of mesophilic and thermophilic lactobacilli, mesophilic and thermophilic cocci, NSLAB and yeasts. Colonies were purified by repetitive streaking, tested for Gram straining, stored at −80 °C in nutrient broth (Biokar, Beauvais, France) supplemented with 20% (*v*/*v*) glycerol (Fluka Analytical, St. Gallen, Switzerland) and subjected to molecular identification.

### 2.3. Identification of Isolates

Bacteria genomic DNA was extracted from overnight cultures using the GenElute^TM^ Bacterial Genomic DNA Kit (NA2110-1KT; Sigma-Aldrich, Darmstadt, Germany). For yeasts, genomic DNA was extracted according to Kazou et al. [25]. DNA concentration of all isolates was measured using a Quawell Q5000 Read First photometer (Quawell Technology Inc., San Jose, CA, USA).

Repetitive extragenic palindromic elements PCR (rep-PCR) was then performed [25] and bacteria and yeasts rep-PCR patterns were separated by electrophoresis and visualized in the transilluminator UVP GelDoc-It Imager (Analytik Jena GmbH, Jena, Germany). Afterwards, BioNumerics software v.6.0 (Applied Maths, Ghent, Belgium) was used for the construction of bacteria and yeasts dendrograms using the unweighted pair group method with arithmetic mean (UPGMA) for clustering.

Subsequently, representative bacteria and yeast isolates based on the clustering were identified at the species level by sequencing the 16S rRNA gene and the ITS DNA region, respectively, using the primer pairs and PCR conditions described previously [25].

### 2.4. Total DNA Extraction and Amplicon Sequencing

Microbial DNA from the pasteurized milk samples used for cheese making was extracted using the DNeasy^®^PowerFood^®^ Microbial Kit (Qiagen, Valencia, CA, USA), whereas for Mana Kopanisti and Kopanisti cheese samples during ripening (Day 1, 7, 21 and 35) DNeasy^®^ PowerSoil^®^ Pro Kit (Qiagen) was applied. DNA elution, determination of DNA concentration and quality as well as storage were performed accordingly [25].

Barcoded amplicon sequencing (bTEFAP^®^) was performed at Molecular Research DNA (MR DNA, Shallowater, TX, USA) on the Illumina MiSeq sequencing platform following the manufacturer’s instructions. Primer pair 27F (5′-AGR GTT TGA TCM TGG CTC AG-3′)—519R (5′-GTN TTA CNG CGG CKG CTG-3′) was used for the amplification of the V1-V3 hypervariable region of the bacteria 16S rRNA gene and ITS1F (5′-CTT GGT CAT TTA GAG GAA GTA A-3′)—ITS2R (5′-GCT GCG TTC TTC ATC GAT GC-3′) to amplify the ITS1-ITS2 DNA region of yeasts/fungi. The PCR conditions and purification of amplicon products were performed according to Papademas et al. [26]. For quality control, the barcodes and primers were removed, paired-end sequences were merged and, short sequences (<150 bp), sequences with ambiguous base calls and chimeras were removed [26]. Finally, zero-radius operational taxonomic units (zOTUs) were taxonomically classified using the Nucleotide Basic Local Alignment Search Tool (BLASTn) against a curated National Center for Biotechnology Information (NCBI) deriving database.

Raw sequencing data are deposited at the European Nucleotide Archive (ENA) under the study PRJEB52558.

### 2.5. Physicochemical Analysis

Compositional analysis of the commercial pasteurized cow milk used for cheese making was performed using MilkoScan^TM^ FT 120 (Foss Electric, Hilleroed, Denmark). Physicochemical analyses of Mana Kopanisti and Kopanisti cheese samples throughout ripening (Day 1, 7, 21 and 35), were performed as follows. The pH of milk and cheese samples was measured at 25 °C using a pH Meter (827 pH lab, Metrohm Herisau, Switzerland). The % (*w*/*w*) moisture content of cheese samples was measured by heating the samples at 105 °C to constant weight. The % (*w*/*w*) ash, salt and fat content of cheese samples were determined according to the standard methods FIL-IDF 27:1964 [27], ISO 5943:2006/IDF 88:2006 [28] and ISO 3433:2008/IDF 222:2008 [29], respectively. The total nitrogen and protein % (*w*/*w*) content of the cheese samples were determined using the Kjeldahl method (ISO 8968-1:2014/IDF 20-1:2014) [30].

### 2.6. Sensory Evaluation

After 35 days of ripening, cheeses were subjected into a blind organoleptic evaluation by a 12-member non-professional, well-experienced tasting panel familiar with Kopanisti cheese, comprising members of the Laboratory of Dairy Research at the Agricultural University of Athens. Cheese samples were served directly from the sterilized plastic containers used for cheese storage, and each panelist received 3 g of cheese sample in a randomized order. The panel graded appearance (scale 0–10), texture and structure (scale 0–40), with the texture being also evaluated with respect to spreadability and mouthfeel and, finally, aroma (scale 0–25), flavour (scale 0–20) and after-taste (scale 0–5), with flavour being also evaluated with respect to peppery and salty traits.

### 2.7. Bioinformatics and Statistical Analysis

Bacteria and yeasts/fungi diversity analysis was evaluated in R v.4.2.0 using several custom packages [31] along with phyloseq [32] and ggplot2 [33]. Alpha-diversity analysis was calculated using the Observed species and the inverse Simpson index values for community diversity according to the abundance and uniformity of zOTUs. For beta-diversity, a hierarchical clustering dendrogram combining Complete as clustering algorithm and Bray–Curtis as distance measure was performed on the zOTUs taxonomically assigned at the genus level. Furthermore, partial least squares discriminant analysis (PLS-DA) and heatmaps of the top 25 bacteria and yeasts/fungi zOTUs using the Ward clustering algorithm and Euclidean distance measure were conducted using Metaboanalyst v.5.0 [34].

Moreover, multiple sample comparison was performed with one-way analysis of variance (ANOVA) followed by the post-hoc Tukey’s Honestly Significant Difference (HSD) test, as those are implemented in STATGRAPHICS Centurion XV (StatPoint Technologies Inc., Warrenton, VA, USA). The same analysis was also used to compare the intra samples diversity estimation (alpha-diversity). Values of *p* < 0.05 were considered to be statistically significant.

## 3. Results and Discussion

### 3.1. Microbiological Analysis

The microbial groups enumerated in the present study are shown in Supplementary Table S1. Interestingly, none of these groups were detected in Mana Kopanisti, while counts in the pasteurized cow milk used were low, ranging from below the detection limit to 3.7 ± 0.7 log CFU mL^−1^.

Considering the microbiological profile of the four Kopanisti types, mesophilic lactobacilli, thermophilic lactobacilli, mesophilic cocci, thermophilic cocci and NSLAB were determined at high levels, as expected. Generally, no statistically significant differences among the four Kopanisti types were observed throughout ripening, apart from Kopanisti D, and, in particular, in the case of mesophilic and thermophilic lactobacilli, thermophilic cocci, NSLAB and yeasts. Occasional statistically significant differences during ripening per Kopanisti type were observed. Interestingly, in Kopanisti D slightly lower numbers of mesophilic and thermophilic lactobacilli, as well as NSLAB were determined.

It is worth noting that counts of both enterococci and enterobacteria were very low, indicating the good hygienic conditions prevailing during cheese making. On the other hand, significant counts were determined for psychrotrophic bacteria; however, we have not isolated colonies from the MPCA medium to proceed with the identification. Finally, yeast counts were at considerable levels throughout ripening, a fact that reflects the common Kopanisti microbiota.

So far, there are limited and rather old literature reports on the microbial groups in Kopanisti. In fact, only Kaminarides and Anifantakis [2] and Kaminarides et al. [6] have dealt with the evolution of the Kopanisti microbiota during ripening, while three other studies have explored the microbiological profile of commercial or artisanal Kopanisti samples [3,8,10]. The microbiota evolution during ripening (ranging from 32 to 46 days) in Kopanisti, which was produced either with the addition of Mana Kopanisti as the sole inoculum [2,6] or using selected yeast/molds strains as starters [6], was similar to our results regarding the microbial groups enumerated by the authors, i.e., total mesophilic bacteria, streptococci, lactobacilli, coliforms and yeasts. This is also valid for the levels of total mesophilic bacteria, LAB, coliforms and yeasts in the majority of the 50 Kopanisti samples collected from retail shops in four islands and Peloponnese over a period of five months [10]. On the contrary, lower counts have been reported by Rhoades et al. [8] for total mesophilic bacteria, LAB, lactic streptococci and yeasts in three Kopanisti samples, produced from pasteurized cow milk without the addition of starters in the islands of Mykonos, Tinos and Siros, with only enterococci being enumerated at higher levels. Finally, the ecosystem of a 3-month-old Kopanisti, artisanally produced in Tinos island without the addition of starters, was quite unusual [3]. The highest LAB counts were obtained for NSLAB (5.9 ± 0.1 log CFU g^−1^), followed by mesophilic lactobacilli, leuconostocs and thermophilic cocci, while no yeasts and coliforms were found.

### 3.2. Identification of Isolates

Based on colony morphology, 17 bacteria were isolated from the pasteurized milk used for cheese production and 45, 44, 45 and 38 cheese samples from Kopanisti A, B, C and D, respectively, were monitored throughout ripening (Day 1, 7, 21 and 35). In addition, 11, 8, 8 and 9 yeast isolates were selected from Kopanisti A, B, C and D, respectively. Rep-PCR analysis clustered bacteria and yeasts isolated from the Kopanisti cheese samples, as shown in Supplementary Figure S1, and representative isolates of all groups were subjected to 16S rRNA gene and ITS DNA region sequencing, respectively.

According to the sequencing results (Supplementary Table S2A), all bacteria isolated from the pasteurized milk were LAB and belonged to the species *L. lactis*, *Leuconostoc lactis*/*garlicum*, *Lactococcus raffinolactis*, *Lacticaseibacillus casei*/*paracasei*/*rhamnosus*, *Streptococcus thermophilus*, *Leuconostoc mesenteroides*, *Streptococcus gallolyticus*/*macedonicus*. In Kopanisti A–C samples, the starters, i.e., *L. lactis* and *Lc. paracasei*, were the dominant isolated species. In addition to the starters, *S. thermophilus*, *Levilactobacillus brevis* and *Lactiplantibacillus plantarum*/*paraplantarum*/*pentosus* were also detected but in small proportions. On the other hand, in Kopanisti D the starters, i.e., *Lig. acidipiscis* and *Loig. rennini*, were not detected, and the isolates belonged to the species *Weissella viridescens*, *Lact. casei*/*paracasei*/*rhamnosus*, *S. thermophilus*, *Lact. plantarum*/*paraplantarum*/*pentosus*, *Lv. brevis* and *Pediococcus pentosaceus*, indicating a higher biodiversity compared to the other Kopanisti types. It should be stressed that *S. thermophilus* and *Lact. casei*/*paracasei*/*rhamnosus* were also detected in pasteurized milk used for cheese production. Therefore, we can assume that these species were most probably transferred from the milk to the cheese microbiota and prevailed during cheese ripening, while *Lig. acidipiscis* and *Loig. rennini*, known as weak milk acidifiers, most probably could not thrive in this environment. Since no yeasts were used as starters to control yeast microbiota, the number of yeast species isolated from the Kopanisti cheeses was higher compared to that of bacteria (Supplementary Table S2B). In brief, the main species detected in all cheese samples were *Torulaspora delbrueckii*, *Rhodotorula mucilaginosa*, *Kluyveromyces marxianus*/*lactis*, *Yarrowia lipolytica*, *Pichia fermentans* and *Pichia membranifaciens*, all commonly reported in the literature regarding cheese microbiota [35,36,37,38,39].

As mentioned before, although there are limited reports on the microbiota of Kopanisti and Mana Kopanisti, our results were broadly in agreement with those of other authors. Regarding bacteria, lactobacilli species e.g., *Lact. plantarum*, *Lev. brevis*, *Lact. casei*/*paracasei*/*rhamnosus*, and *Lactobacillus delbrueckii*, were the most commonly isolated ones, while in some cases, enterococci have been also found [8,9,10]. Regarding yeasts, only two studies [2,10] have been dealt with so far and species, such as *P. membranifaciens*, *P. fermentans*, *Candida kefyr*, *Kluyveromyces* sp., *Trichosporon cutaneum*, *K. lactis*, *Saccharomyces exiguous*, *Rhodotorula rubra*, *Saccharomyces cerevisiae*, *Trichosporon penicillatum*, *Candida lusitaniae* and *Debaryomyces hansenii*, have been identified. Interestingly, Asteri et al. [3], isolated from Kopanisti and Mana Kopanisti only two species, namely *Lig. acidipiscis* and *Loig. rennini*, and well-studied isolates [18,19,20] have been used in the present study.

### 3.3. Metataxonomics Analysis

#### 3.3.1. Sequencing Data and Alpha Diversity Analysis

A total of 5,332,991 raw 16S sequences were obtained from the pasteurized milk used for cheese production, Mana Kopanisti and Kopanisti cheese samples throughout ripening (Day 1, 7, 21 and 35). After the quality control, 1,321,909 sequences were used for taxonomic classification, with an average of 24,630 ± 1956 sequences per milk sample and 23,988 ± 1257 per cheese sample (Mana Kopanisti and Kopanisti). In addition, 473 bacterial zOTUs were assigned among the samples, with the average number of zOTUs being 413 ± 12 for milk samples and 166 ± 74 for Mana Kopanisti and Kopanisti cheese samples. The number of yeast/fungal raw sequences obtained from all samples analyzed was similar to that of bacterial sequences, i.e., 5,961,637. However, the number of sequences that passed the quality control was much higher compared to bacteria, i.e., 4,603,637 with an average of 12,157 ± 5776 sequences per milk sample and 89,313 ± 81,518 per cheese sample (Mana Kopanisti and Kopanisti). Nevertheless, the number of yeast/fungal zOTUs was considerably less than that of bacteria, i.e., 158, with the average number of zOTUs being 107 ± 12 for milk samples and 166 ± 74 for Mana Kopanisti and Kopanisti cheese samples.

**Figure 1:**
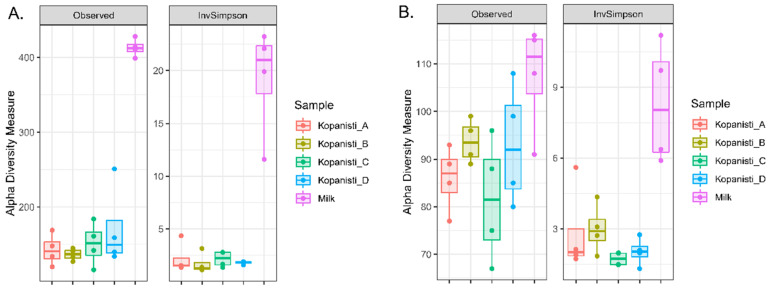
Alpha-diversity boxplots for (**A**) bacteria and (**B**) yeasts/fungi communities identified in pasteurized milk and Kopanisti A, B, C and D cheese samples. Statistically significant differences (*p* < 0.05) were detected between each Kopanisti type and pasteurized milk for inverse Simpson indices (both bacteria and yeasts/fungi) and for Observed index (only for bacteria). Regarding the Observed index, statistically significant difference (*p* < 0.05) was only detected between Kopanisti C and pasteurized milk.

The Observed and inverse Simpson indices were used to evaluate the microbial complexity (richness and evenness) of pasteurized milk, Kopanisti A, B, C and D cheese samples throughout ripening. The richness estimation (Observed index) indicated that there were no differences in the bacteria microbiota among Kopanisti cheese samples, whereas a significant difference (*p* < 0.05) was observed between the milk samples and each Kopanisti cheese sample (Figure 1A). Similarly, a significant difference (*p* < 0.05) was only found between the milk and Kopanisti samples according to the inverse Simpson index (both richness and evenness). On the other hand, the yeast/fungi communities were found to be more in balance among the different samples analyzed, as the only significant difference (*p* < 0.05) was observed between milk and Kopanisti C samples (Figure 1B). However, this was not the case for inverse Simpson index, as the diversity of the yeast/fungal microbiota of milk samples was significantly higher (*p* < 0.05) compared to the Kopanisti cheese samples.

#### 3.3.2. Bacteria Communities in Pasteurized Milk, Mana Kopanisti and Kopanisti Cheese Samples

Both bacteria and yeasts/fungi taxa of all samples analyzed were assessed up to the genus level for a more accurate identification, due to the high-level similarity between closely-related taxa. This is important especially in the case of yeasts/fungi, as cut-off equivalents for genus or species levels are quite difficult [40].

**Figure 2:**
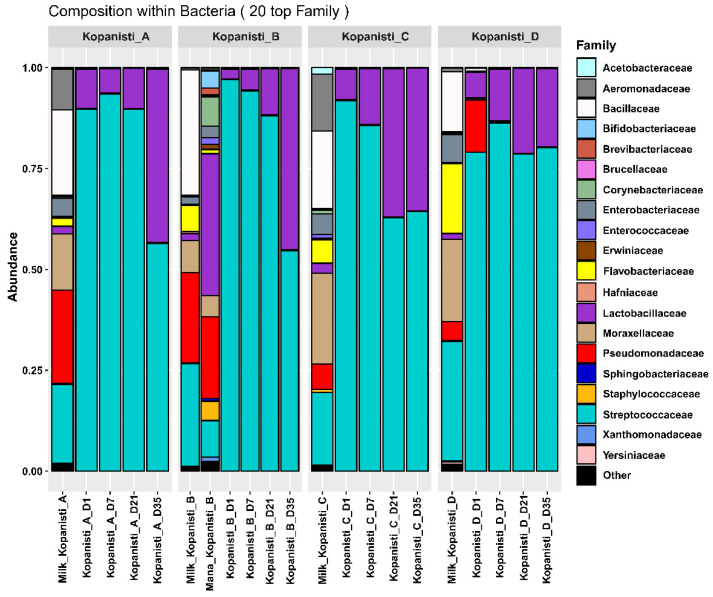
Composition plots of the relative abundances of the 20 most abundant bacteria zOTUs taxonomically assigned at the family level in pasteurized milk, Mana Kopanisti and Kopanisti cheese samples. Samples from each Kopanisti type (A–D) are presented together in subpanels.

As it was expected, significantly more families were detected in milk samples compared to the Kopanisti cheese samples, as the addition of starters results in a more balanced cheese microbiota (Figure 2) [22,41,42]. The most abundant families detected in milk samples were *Streptococcaceae*, *Pseudomonadaceae*, *Moraxellaceae*, *Bacillaceae*, *Aeromonadaceae* and *Flavobacteriaceae*. Interestingly, the bacteria microbiota of Mana Kopanisti was also highly diverse and more similar to that of milk compared to the Kopanisti cheese samples. On the other hand, no matter the Kopanisti type, *Streptococcaceae* and *Lactobacillaceae* were the predominant families identified throughout ripening. However, it should be noted that on Day 1 of Kopanisti D, *Pseudomonadaceae* was also identified in relatively high abundance. This family seems to have been transferred from the milk to Kopanisti D Day 1 sample but eliminated during ripening, and thus, did not compromise the safety of the final product. Notably, although *Pseudomonadaceae* was also detected in the other milk samples, the addition of *L. lactis* and *Lc. paracasei* as starters in Kopanisti A–C samples inhibited the growth of *Pseudomonadaceae.*

**Figure 3:**
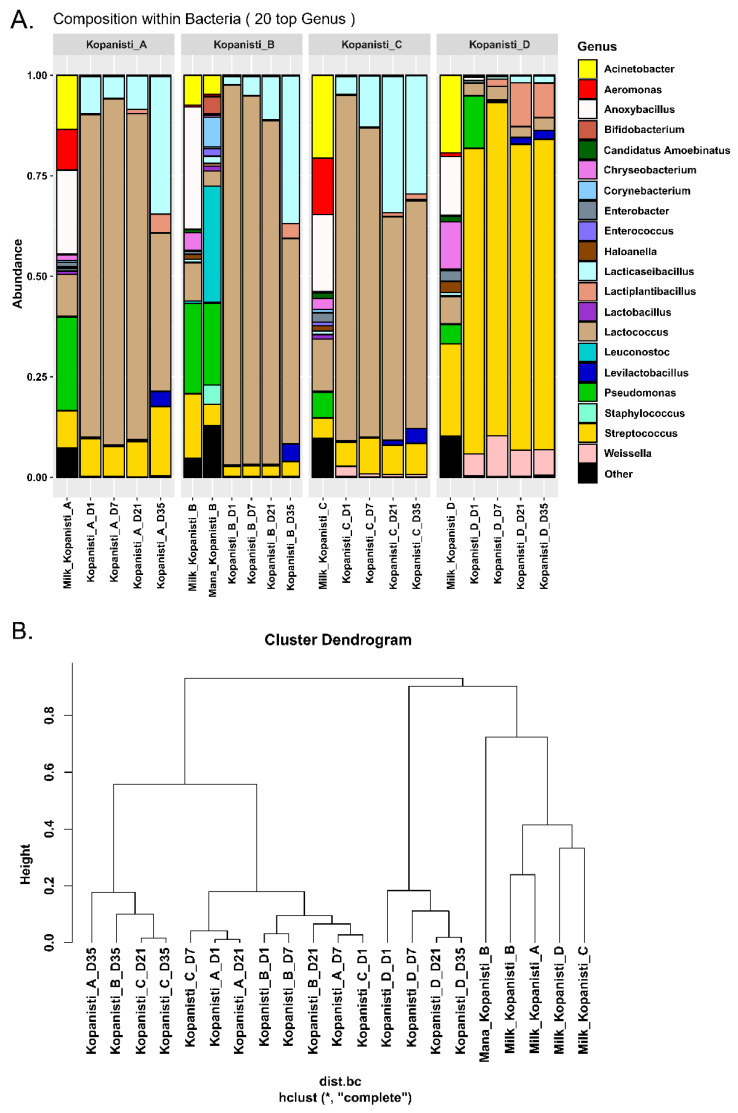
Composition plots of the relative abundances of the 20 most abundant bacteria zOTUs taxonomically assigned at the genus level in pasteurized milk, Mana Kopanisti and Kopanisti cheese samples. Samples from each Kopanisti type (A–D) are presented together in subpanels (**A**). Hierarchical clustering of the bacteria communities at the genus level identified in pasteurized milk, Mana Kopanisti and Kopanisti cheese samples (**B**).

The similarities of the bacteria microbiota at the family level observed among the Kopanisti A–C samples were also observed at the genus level. In brief, *Lactococcus* and *Lacticaseibacillus* were the main genera identified, as also revealed by the classical microbiological analysis (Supplementary Table S2A), due to the addition of *L. lactis* and *Lc. paracasei* as starters. In addition, *Streptococcus* was also found in relatively high abundance, which was most probably derived from the milk (Figure 3A). Of note, Mana Kopanisti did not appear to shape the bacteria communities of Kopanisti B, not even on Day 1. This was in agreement with the results of the classical microbiological analysis, as none of the microbial groups tested were detected in Mana Kopanisti. However, metataxonomics analysis identified several genera in Mana Kopanisti, e.g., *Leuconostoc*, *Pseudomonas*, *Lacticaseibacillus*, *Corynebacterium*, *Streptococcus* and *Acinetobacter*, indicating that the relative abundances of these genera derived from the amplification of dead or compromised cells [43]. The hypothesis of a transferred microbiota from the milk to the Kopanisti D cheese, which was initially suggested based on the classical microbiological analysis, was strengthened by the results of the metataxonomics analysis. In brief, the main genera detected in Kopanisti D, i.e., *Streptococcus*, *Weissella*, *Lactiplantibacillus*, *Lactococcus* and *Pseudomonas*, seems to be conveyed from milk. Once again, the metataxonomics results were consistent with those of the classical microbiological analysis, since the majority of the isolated bacteria from Kopanisti D belonged to the genera *Streptococcus*, *Weissella* and *Lactiplantibacillus* (Supplementary Table S2A).

As shown in the hierarchical clustering of the bacteria genera identified in all samples analyzed, two main clusters were formed (Figure 3B). The first consisted of Kopanisti A–C samples and the second of Kopanisti D, Mana Kopanisti and milk samples. This clustering supports the results of both classical microbiological analysis (Supplementary Table S2A) and compositional analysis (Figure 3A) presented above as well as the hypothesis that milk microbiota was shifted to Kopanisti D. The subgroups in the first cluster indicate that neither sample type (Kopanisti A–C) nor sampling point (Day 1–35) were important factors to discriminate the samples. On the contrary, the subgroups formed in the second main cluster revealed that the samples from the same type, i.e., Kopanisti D, Mana Kopanisti and milk, grouped together under the same branch. Specifically, for Kopanisti D, these samples formed three groups with the sample on Day 1 being the most distant due to the presence of the genus *Pseudomonas*, as mentioned before. Moreover, samples on Days 21 and 35 clustered together due to their similar microbiota (genera identified and relative abundances), indicating that the bacteria microbiota from Day 21 until the end of ripening process was stable without significant differences occurred.

**Figure 4:**
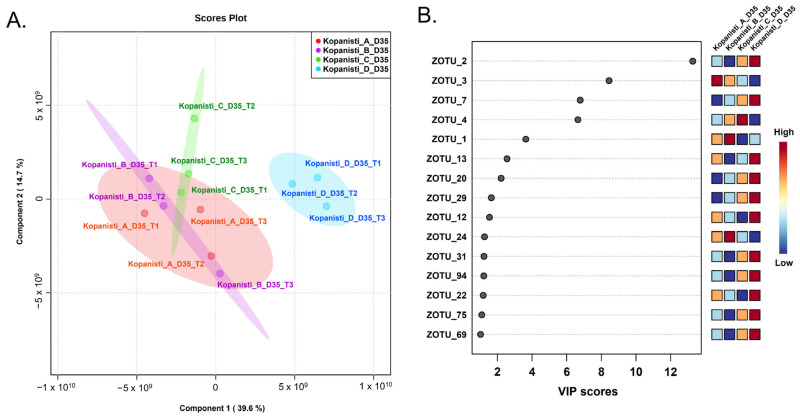
Partial least squares discriminant analysis (PLS-DA) of the bacteria zOTUs detected in the Kopanisti A, B, C and D samples on Day 35. Samples are coloured according to the Kopanisti type, i.e., red for Kopanisti A, purple for Kopanisti B, green for Kopanisti C and blue for Kopanisti D (**A**). Most influential bacteria zOTUs of the Kopanisti A, B, C and D samples on Day 35 were based on the Variable Importance in Projection (VIP) scores from the PLS-DA. The colour bar indicates the intensity of the variable in the respective Kopanisti type (**B**).

To further explore the degree of diversity among the different Kopanisti types (A–D), a PLS-DA analysis was performed only for the final products (Day 35) using the zOTUs identified in all three trials performed for each Kopanisti type. As shown in Figure 4A, PLS-DA analysis also supported the discrimination of Kopanisti D, as the other three Kopanisti types grouped together, due to the addition of the same starters. This clustering indicates the strong impact of *L. lactis* and *Lc. paracasei* to the formation of the bacteria microbiota regardless of the addition of Mana Kopanisti in Kopanisti B or the inclusion of *Lig. acidipiscis* and *Loig. rennini* as adjuncts in Kopanisti C. According to the Variable Importance in Projection (VIP) scores, seven zOTUs were considered as highly influential for the clustering resulted by PLS-DA (Figure 4B). In details, zOTU_2 (*Streptococcus thermophilus*), zOTU_7 (*Weissella viridescens*), zOTU_13 (*Lactiplantibacillus plantarum*) and zOTU_20 (*Weissella viridescens*) were highly associated with Kopanisti D, zOTU_4 (*Lacticaseibacillus paracasei*) with Kopanisti C, zOTU_1 (*Lactococcus lactis*) with Kopanisti B and zOTU_3 (*Lacticaseibacillus paracasei*) with Kopanisti A. VIP scores are in agreement with those by both classical microbiological analysis (Supplementary Table S2A) and compositional analysis (Figure 3A) presented above, as the starters, either *L. lactis* or *Lc. paracasei*, seems to influence the microbiota of Kopanisti A–C on Day 35. On the other hand, the zOTUs found to be highly associated with Kopanisti D on Day 35 once again were not the starters, but species originated either from the milk or the environment during cheese making and ripening.

To our knowledge, this is the first metataxonomics study evaluating the microbiota of Kopanisti cheese throughout production and ripening. There is only one study that has monitored the bacteria and yeasts/fungi communities of three Kopanisti samples, among five other Greek PDO cheeses, but these samples were final products purchased from producers or industries [44]. The bacteria microbiota of these samples, originating from Tinos, Siros and Mykonos islands, were mainly dominated by the genera *Lactobacillus*, *Streptococcus*, *Leuconostoc* and *Serratia*, which were in line with our results. Apart from Kopanisti, metataxonomics has also been used to evaluate the bacteria microbiota of several cheese types, e.g., Feta, Graviera, Kaseri, Halloumi, Halitzia, Kefalotyri, Serrano, Edam, Cheddar, Gouda, Brie, Saint-Marcellin and Maroilles, with the main genera identified being *Lactococcus*, *Streptococcus*, *Lactobacillus*, *Leuconostoc*, *Pediococcus* and *Weissella* [26,44,45,46,47,48,49,50,51,52]. However, the significance of their occurrence was not investigated, and thus, further studies are needed.

As it was clearly revealed by both our results and those of other metataxonomics studies, LAB taxa prevail in the cheese ecosystem. However, it should be noted that apart from LAB, spoilage or potential pathogenic bacteria can be also detected especially via culture-independent analysis. In our samples, no reads from well-known foodborne pathogens, such as *Staphylococccus aureus*, *Listeria monocytogenes*, *Bacillus cereus*, *Salmonella* spp. and *Clostridium* spp., were detected. However, few reads (≤3) were taxonomically assigned to *Escherichia coli*, *Shigella spp.*, *Streptococcus uberis*, *Streptococcus agalactiae*, *Streptococcus dysgalactiae* and *Bacillus thuringiensis* in the final Kopanisti cheese products (Day 35) (Supplementary Table S3). However, metatranscriptomics needs to be performed to investigate whether these bacteria are active or not. It should be noted though, that the presence of spoilage or potential pathogenic taxa in cheeses has also been reported in other metataxonomics studies [44,46,47,50,52,53,54,55].

#### 3.3.3. Yeasts/Fungi Communities in Pasteurized Milk, Mana Kopanisti and Kopanisti Cheese Samples

**Figure 5:**
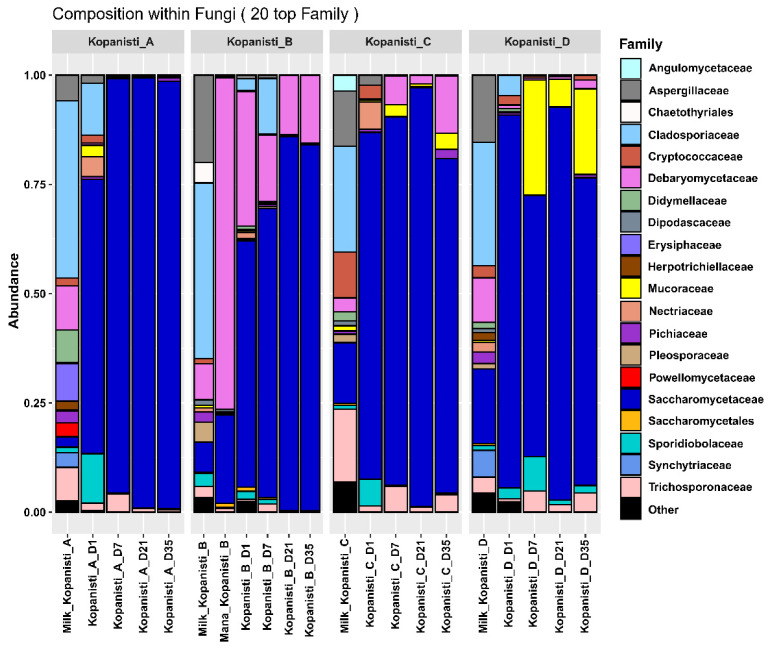
Composition plots of the relative abundances of the 20 most abundant yeasts/fungi zOTUs taxonomically assigned at the family level in pasteurized milk, Mana Kopanisti and Kopanisti cheese samples. Samples from each Kopanisti type (A–D) are presented together in subpanels.

The different yeast/fungi microbiota at the family level of pasteurized milk, Mana Kopanisti and Kopanisti cheese samples throughout ripening are presented in Figure 5. In brief, *Cladosporiaceae*, *Aspergillaceae*, *Debaryomycetaceae*, *Saccharomycetaceae* and *Trichosporonaceae* were among the most abundant families in milk samples. Regarding Mana Kopanisti, although its bacteria microbiota was more similar to that of milk samples, as mentioned before, in the case of yeasts/fungi, Mana Kopanisti was found to be more similar to Kopanisti B. Therefore, it seems that the yeasts/fungi microbiota of Mana Kopanisti influenced the microbiota of Kopanisti B, even though this was not the case for bacteria. This may be assigned to the starters that were added that dominated the bacteria microbiota of Kopanisti B. Regarding the families identified in both Mana Kopanisti and Kopanisti B, *Saccharomycetaceae* and *Debaryomycetaceae* were the most abundant. In addition, *Saccharomycetaceae* was found to be the dominant family detected in Kopanisti A, whereas on Day 1, *Sporidiobolaceae*, *Cladosporiaceae* and *Nectriaceae* were also identified in high abundances. Overall, the microbiota of Kopanisti A remained practically stable from Day 7 until the end of ripening. On the contrary, the yeasts/fungi microbiota of Kopanisti C continue to evolve during ripening, with the majority of the families identified being the same as in Kopanisti A, though in different abundances. This suggests that the addition of the adjuncts affected the microbiota of the Kopanisti C samples. Of note, *Mucoraceae*, which was identified in relatively low abundances in the majority of milk and Kopanisti A–C samples, was found to be the second most abundant family in Kopanisti D samples, indicating that most probably the addition of starters in the Kopanisti A–C samples impeded *Mucoraceae* from prevailing.

**Figure 6:**
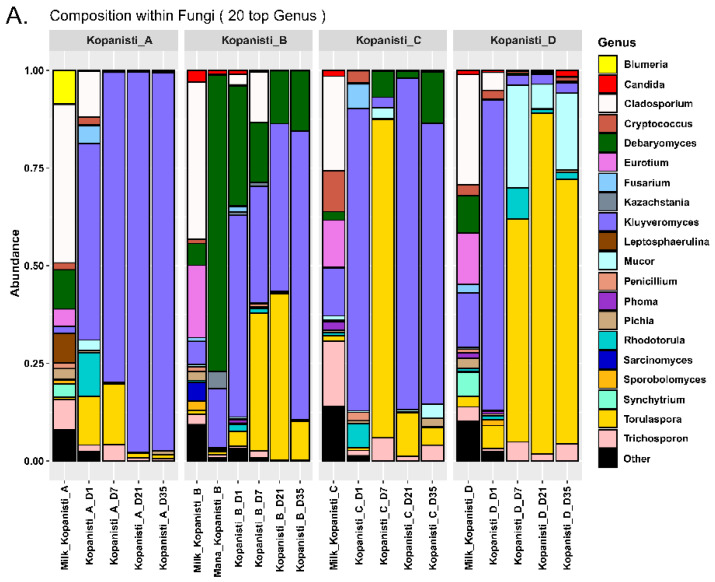

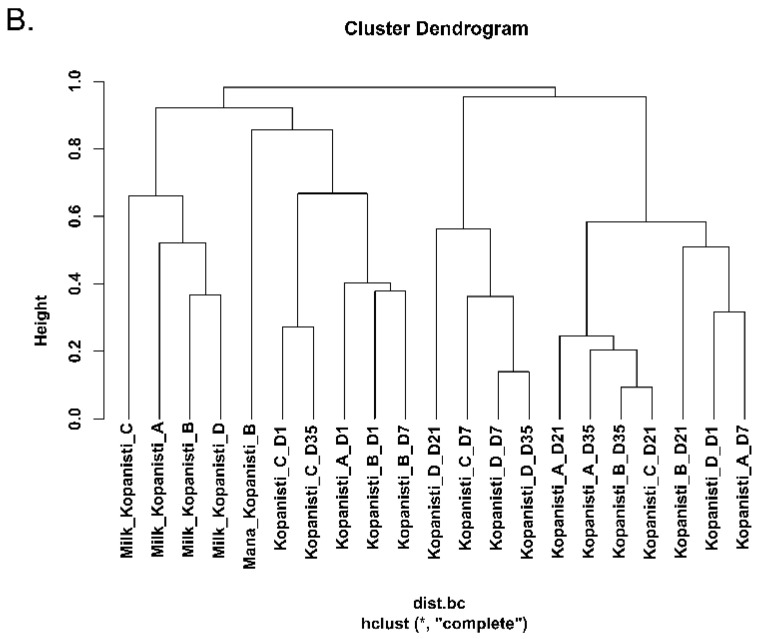
(**A**) Composition plots of the relative abundances of the 20 most abundant yeasts/fungi zOTUs taxonomically assigned at the genus level in pasteurized milk, Mana Kopanisti and Kopanisti cheese samples. Samples from each Kopanisti type (A–D) are presented together in subpanels. (**B**) Hierarchical clustering of the yeasts/fungi communities at the genus level identified in pasteurized milk, Mana Kopanisti and Kopanisti cheese samples.

At the genus level, *Kluyveromyces*, *Debaryomyces* and *Torulaspora* were mainly identified in Mana Kopanisti and Kopanisti A–C samples (Figure 6A). Despite the similar genera detected in these samples, their abundances varied based on the sample analyzed; however, the pattern was comparable to that of families. In brief, the microbiota of Kopanisti A samples was more stable than B and C samples, and Mana Kopanisti seems to influence the microbiota of Kopanisti B, although this did not happen in bacteria, as mentioned before. Regarding Kopanisti C, although *Kluyveromyces* was the main genus detected, on Day 7 *Torulaspora* prevailed. However, the abundance of *Torulaspora* significantly decreased from Day 21 until the end of ripening. In contrast to the other Kopanisti types, the microbiota of Kopanisti D was more diverse, as the number of genera detected was much higher. However, from Day 7 the microbiota stabilized, with *Torulaspora* and *Mucor* being the main genera identified. The genera detected through metataxonomics analysis were consistent with the results from the classical microbiological analysis (Supplementary Table S2B). In brief, the yeasts isolated from the Kopanisti samples during culture-dependent analysis belonged to the genera *Kluyveromyces*, *Torulaspora*, *Rhodotorula*, *Trichosporon*, *Pichia* and *Candida*, all being among the 20 top genera identified in metataxonomics (Figure 6A).

**Figure 7:**
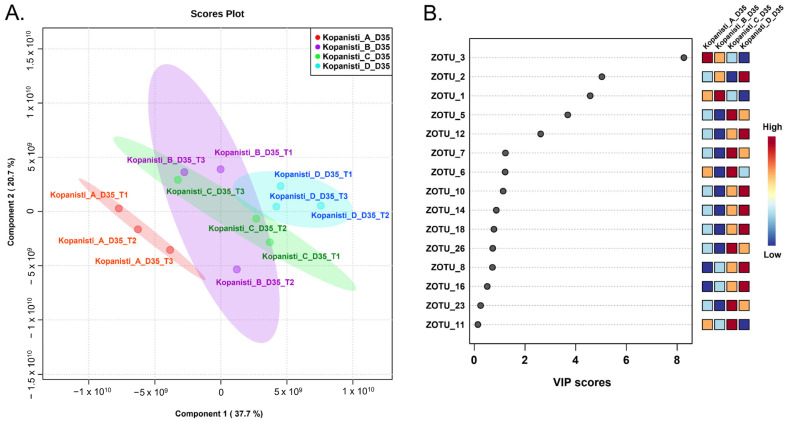
Partial least squares discriminant analysis (PLS-DA) of the yeasts/fungi zOTUs detected in the Kopanisti A, B, C and D samples on Day 35. Samples are coloured according to the Kopanisti type, i.e., red for Kopanisti A, purple for Kopanisti B, green for Kopanisti C and blue for Kopanisti D (**A**). Most influential yeasts/fungi zOTUs of the Kopanisti A, B, C and D samples on Day 35 based on the Variable Importance in Projection (VIP) scores from the PLS-DA. The colour bar indicates the intensity of the variable in the respective Kopanisti type (**B**).

In contrast to the bacteria hierarchical clustering (Figure 3B), the yeasts/fungi one based on the genera identified did not have a similar clustering. Apart from the milk samples that grouped together, Kopanisti samples were not clustered either by type (A–D) or sampling point (Day 1–35). This indicates that neither of these two parameters was important for the formation of the yeasts/fungi microbiota, and thus, the inoculation schemes used did not differentiate the Kopanisti types. In order to see whether the Kopanisti final products were separate, we employed PLS-DA analysis among Kopanisti A–D cheese samples on Day 35 using the zOTUs identified in all three cheese-making trials. As shown in Figure 7A, Kopanisti A and D samples created two separate groups, while, on the other hand, an overlap was observed among Kopanisti B and C samples. This indicates that the addition of only *L. lactis* and *Lc. paracasei* differentially influenced the yeasts/fungi microbiota of the final product of Kopanisti A compared to Kopanisti B and C, where Mana Kopanisti and *Lig. acidipiscis*/*Loig. rennini* were additionally used, respectively. In the case of Kopanisti D, PLS-DA analysis of both bacteria and yeasts/fungi zOTUs separated the final products, implying that the microbiota of Kopanisti D was significantly different compared to the other three types. Based on the VIP scores of PLS-DA (Figure 7B), zOTU_3 (*Kluyveromyces marxianus*) was highly associated with Kopanisti A, and zOTU_2 (*Torulaspora delbrueckii*) and zOTU_12 (*Mucor circinelloides*) with Kopanisti D, as also revealed by the compositional analysis at the genus level (Figure 6A).

Although yeasts/fungi play a crucial role in many cheese varieties, metataxonomics studies regarding the yeast/fungi communities in cheese are quite fewer compared to the bacteria ones. Michailidou et al. [44] analyzed three Kopanisti cheese samples from Tinos, Siros and Mykonos islands, and, among the genera identified, *Candida*, *Debaryomyces*, *Kluyveromyces* and *Torulaspora* were among the most abundant, while a relatively high percentage of reads could not be identified, especially in the two of the three samples. These genera also prevailed in the Kopanisti cheese samples analyzed in our study. Regarding other cheese types, e.g., Feta, Galotiri, Halitzia, Serrano, Edam, Cheddar, Gouda and Brie, Tomme d’Orchies, Serpa, Saint-Marcellin and Maroilles, metataxomics revealed that the majority of the taxa belonged to the genera *Debaryomyces*, *Kluyveromyces*, *Pichia*, *Candida*, *Penicillium* and *Trichosporon* [26,44,46,48,51,52,56,57]. However, as in the case of bacteria, further studies are required to evaluate the significance of their occurrence in overall cheese quality.

Regarding the spoilage fungi detected in our cheese samples, only *Mucor circinelloides* was found in relative high reads (Supplementary Table S4), which is responsible for the fungal infection mucormycosis. In addition, *Candida parapsilosis* and *Penicillium commune* were also found, but in only one and less than nine reads, respectively, in the final Kopanisti cheese samples (Day 35). However, as in the case of bacteria, we do not know if these reads have been amplified from alive or dead/compromised cells. It should be also mentioned that these spoilage fungi have also been identified using metataxonomics analysis in several cheese samples [44,46,52,56,58,59,60,61].

### 3.4. Physicochemical Analysis

The composition of the pasteurized full fat cow milk used for all cheese making trials, as determined by MilkoScan, was as follows: 4.34 ± 0.08% (*w*/*w*) lactose, 3.41 ± 0.12% (*w*/*w*) fat, 3.20 ± 0.06% (*w*/*w*) protein and 12.05 ± 0.17% (*w*/*w*) total solids, while the pH was 6.76 ± 0.03.

In the case of Mana, results were as follows: pH 5.16 ± 0.03; moisture 42.39 ± 0.75 (%, *w*/*w*); salt in moisture 10.12 ± 0.20 (%, *w*/*w*); ash 6.15 ± 0.06 (%, *w*/*w*); FDM 53.44 ± 0.90 (%, *w*/*w*); and protein 22.52 ± 1.32 (%, *w*/*w*). As far as we are aware, this is the first time that these characteristics are reported; however, this was a single Mana Kopanisti sample, and, thus, no conclusions can be drawn.

The results of the physicochemical analyses for all Kopanisti cheese samples are presented in Supplementary Table S5. In general, our results regarding the physicochemical characteristics of Kopanisti cheese are in accordance with those reported in the literature [1,5,6,7,8,10]. No statistically significant differences were observed among the four Kopanisti types during ripening, except for a few, mainly regarding Kopanisti B. In brief, the pH in Kopanisti B and D was higher on Day 1 compared to the other types. This could be explained by the buffering capacity of the Mana Kopanisti added in Kopanisti B, since the curd pH was measured after its addition, and the use of *Lig. acidipiscis* and *Loig. rennini* (both known as weak milk acidifiers, as mentioned before), as starters in Kopanisti D. Furthermore, the low moisture/high salt in moisture and high protein content of Mana Kopanisti also affected the respective characteristics of Kopanisti B, as it was expected, and thus, distinguish this type from the other three, especially on Day 35 (*p* < 0.05).

### 3.5. Sensory Analysis

**Figure 8:**
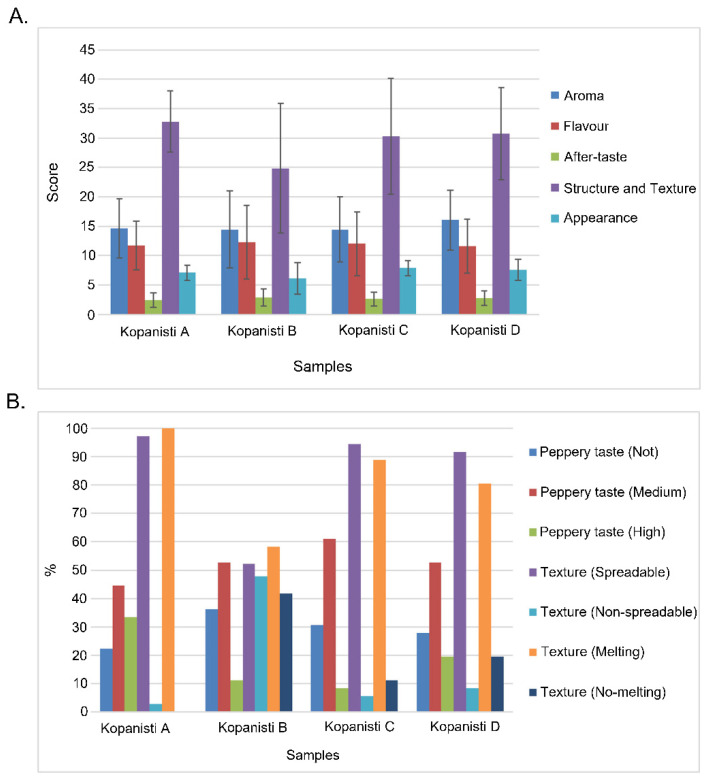
Graphical representation of sensory evaluation of Kopanisti A, B, C and D cheese samples on Day 35 regarding (**A**) aroma, flavour, after-taste, structure and texture and appearance, and (**B**) peppery taste (not/medium/high), texture (spreadable/non-spreadable) and texture (melting/no-melting). Statistically significant differences (*p* < 0.05) were detected between Kopanisti A and B, Kopanisti B and C, and Kopanisti B and D for structure and texture, and between Kopanisti B and C, and Kopanisti B and D for appearance.

The sensory assessment of the Kopanisti cheeses is presented in Figure 8.

Even though no statistically significant differences among the four Kopanisti cheese types were observed in the majority of the sensory traits evaluated, in the case of structure and texture, Kopanisti B received the lowest score (*p* < 0.05). This was also reflected when the spreadability and melting were evaluated, as the two opinions (spreadable/non-spreadable and melting/no-melting) were equivalently evaluated for Kopanisti B compared to the other three types. Therefore, type B is considered the least acceptable, since Kopanisti is a soft cheese, characterized by a smooth texture and good spreadability. In addition, Kopanisti B had also the lowest score in appearance (*p* < 0.05), indicating that the combination of the specific Mana Kopanisti and starter cultures did not result in an accepted product based on the sensory evaluation.

Among the other three types, although not in a statistically significant mode, Kopanisti A received the highest score in structure and texture, Kopanisti C in flavour and appearance and Kopanisti D in aroma and after-taste. Interestingly, even though *Lig. acidipiscis* and *Loig. rennini* could not thrive in cheese environment, it seems that their cell lysis led to the release of intracellular enzymes into the Kopanisti cheese matrix (Kopanisti C and D), and thus to the formation of flavour volatiles through proteins, fats and amino acids degradation. Previous studies performed by our group support this hypothesis on the basis that the necessary enzymes for the production of key volatile/flavour metabolites have been identified, and the majority of these metabolites are degradation products of amino acids [3,18]. Although cell lysis and product flavour relation needs to be deeply investigated, it is nowadays believed that enhanced cell lysis leads to increased flavour formation during cheese ripening [62].

Based on the above, we can assume that the addition of *L. lactis* and *Lc. paracasei* resulted in a typical Kopanisti in the case of structure and texture, while, on the other hand, the addition of *Lig. acidipiscis* and *Loig. rennini*, either as adjuncts or starters, mainly contributed to the aroma and flavour. Therefore, type C seems to have the most appropriate bacteria cocktail for the Kopanisti cheese production (*L. lactis*, *Lc. paracasei*, *Lig. acidipiscis* and *Loig. rennini*).

## 4. Conclusions

In the present study, different bacteria species were used in four inoculation schemes (A–D) to produce Kopanisti cheese, in order to study their impact on the microbial, physicochemical and sensory profile throughout cheese production and ripening, and thus, their ability to be used as suitable starters or adjuncts in a more standardized Kopanisti cheese production that could lead to the final products of high quality and safety. Our study showed that this dual approach, i.e., the employment of both classical microbiological and metataxonomics analyses, is a powerful tool to analyze the microbial communities of fermented foods, including Kopanisti cheese and most importantly, metataxonomics revealed the microbial taxa that distinguished the four batches obtained with the addition of different cultures. In addition, the presence of spoilage and/or potential pathogenic taxa, although in low reads in the final products, underlines the necessity of multi-omics approaches to test whether these microorganisms are active or not, and probably the application of techniques, such as post-pasteurization, to ensure the safety of the final products. In conclusion, the present study highlights the importance of looking for new bacterial cultures for improving the overall quality and safety of cheese products in the market. The combination of both conventional and state-of-the-art methods could provide an integrated approach for an in-depth characterization of the cheese microbial and physicochemical profile.

## Data Availability

Raw sequencing data regarding the 16S and ITS metataxonomics analysis are deposited at the European Nucleotide Archive (ENA) under the study PRJEB52558.

## Supplementary Materials

The following supporting information can be downloaded at: https://www.mdpi.com/article/10.3390/microorganisms11010066/s1, Supplementary Figure S1: rep-PCR fingerprinting of (A, C, E, G) the bacteria isolated from Kopanisti A, B, C and D cheese samples using the BOXAIR primer and (B, D, F, H) the yeasts isolated from Kopanisti A, B, C and D cheese samples using the (GTG)_5_ primer. Image analysis was performed by BioNumerics v.6.0. Red lanes separate the groups based on their similarity and the red bacteria and yeast isolates were selected for 16S rRNA gene and ITS DNA region sequencing, respectively.; Supplementary Table S1: Microbiological evaluation (mean log CFU mL^−1^ or g^−1^ ± SD) of pasteurized milk, Mana Kopanisti and Kopanisti cheese samples during ripening.; Supplementary Table S2: Identification of (A) bacteria and (B) yeasts isolated from pasteurized milk and Kopanisti A, B, C and D cheese samples during ripening (Day 1, 7, 21 and 35). Isolates in red letters have been selected for taxonomic identification at the species level by 16S rRNA gene and ITS DNA region sequencing, respectively.; Supplementary Table S3: Taxonomic identification of zOTU obtained by the 16S metataxonomics analysis and number of reads detected in pasteurized milk, Mana Kopanisti and Kopanisti cheese samples during ripening.; Supplementary Table S4: Taxonomic identification of zOTU obtained by the ITS metataxonomics analysis and number of reads detected in pasteurized milk, Mana Kopanisti and Kopanisti cheese samples during ripening.; Supplementary Table S5: Physicochemical evaluation (mean values ± SD) of Mana Kopanisti and Kopanisti cheese samples during ripening.
