# Supplementary material for: The Impact of Different Inoculation Schemes on the Microbiota, Physicochemical and Sensory Characteristics of Greek Kopanisti Cheese throughout Production and Ripening
Source: Microorganisms. 2022 Dec 26;11(1):66. doi: 10.3390/microorganisms11010066 (PMC9863000; doi:10.3390/microorganisms11010066)
Supplement: Supplementary file 1 [file microorganisms-11-00066-s001.zip › Supplementary Figure S1.pptx]

## Slide 1
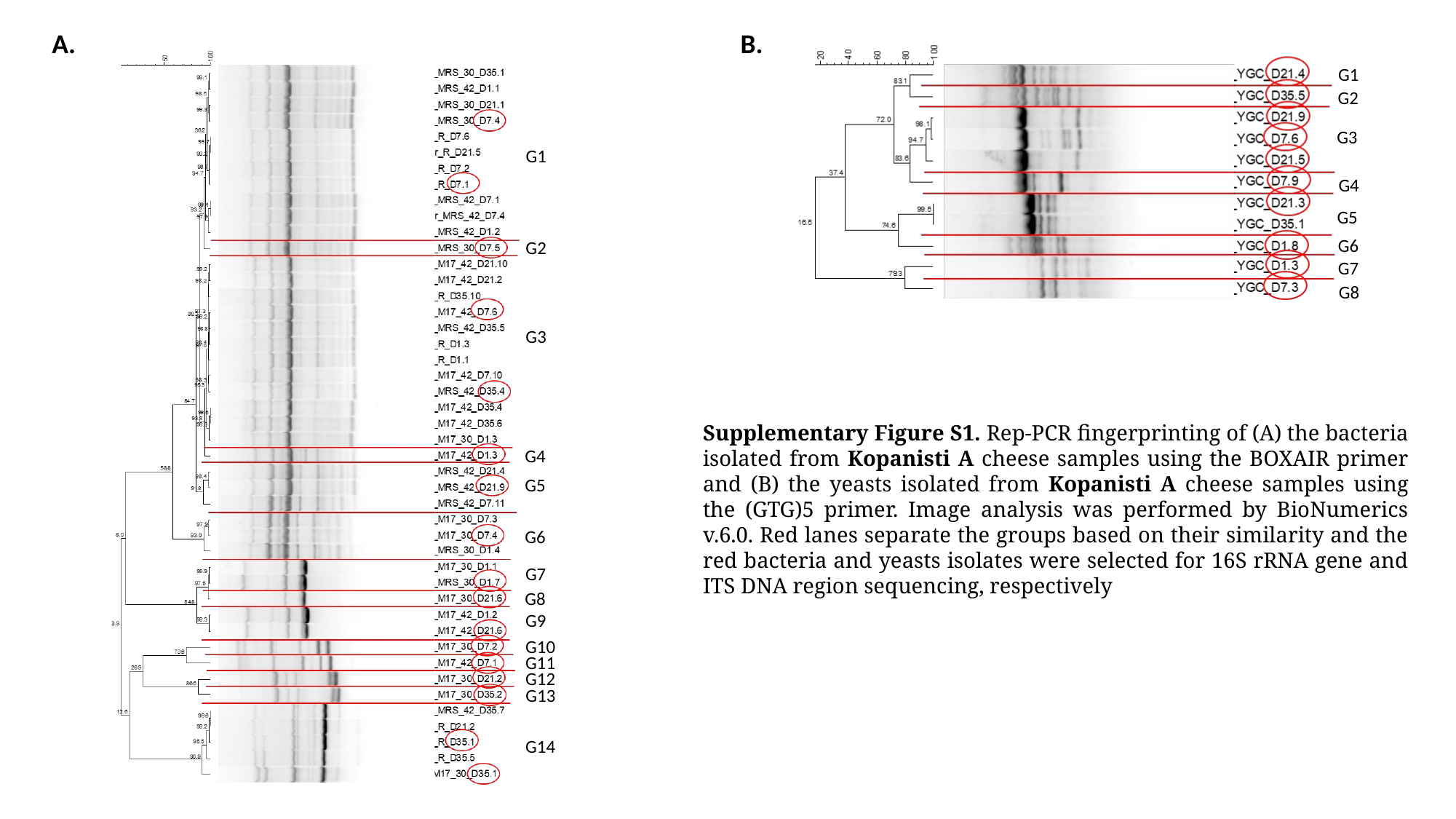

B.
A.
G1
G2
G3
G4
G5
G6
G7
G8
G1
G2
G3
G4
G5
G6
G7
G8
G9
G10
G11
G12
G13
G14
Supplementary Figure S1. Rep-PCR fingerprinting of (A) the bacteria isolated from Kopanisti A cheese samples using the BOXAIR primer and (B) the yeasts isolated from Kopanisti A cheese samples using the (GTG)5 primer. Image analysis was performed by BioNumerics v.6.0. Red lanes separate the groups based on their similarity and the red bacteria and yeasts isolates were selected for 16S rRNA gene and ITS DNA region sequencing, respectively

## Slide 2
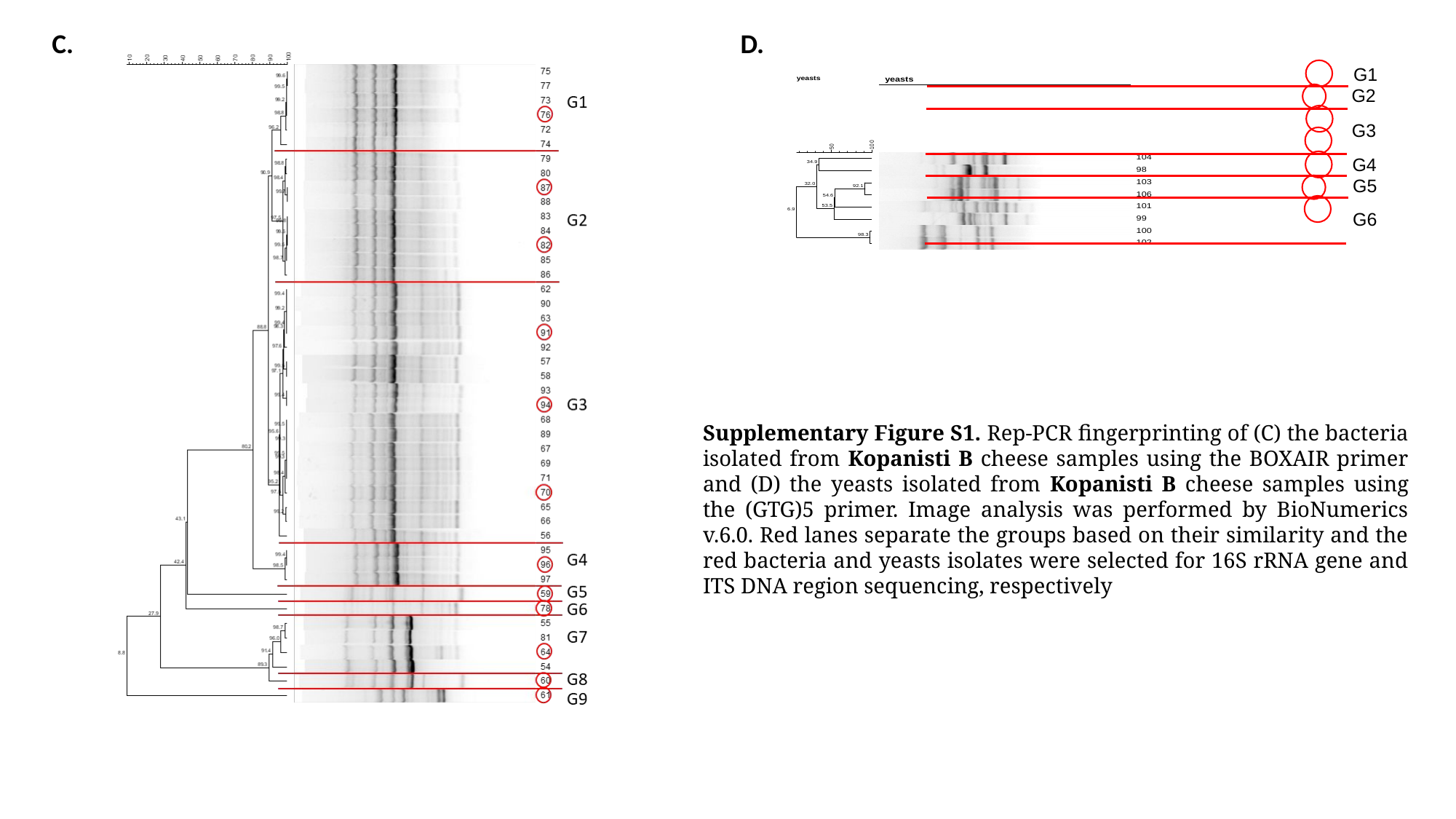

D.
C.
G1
G2
G3
G4
G5
G6
Supplementary Figure S1. Rep-PCR fingerprinting of (C) the bacteria isolated from Kopanisti B cheese samples using the BOXAIR primer and (D) the yeasts isolated from Kopanisti B cheese samples using the (GTG)5 primer. Image analysis was performed by BioNumerics v.6.0. Red lanes separate the groups based on their similarity and the red bacteria and yeasts isolates were selected for 16S rRNA gene and ITS DNA region sequencing, respectively

## Slide 3
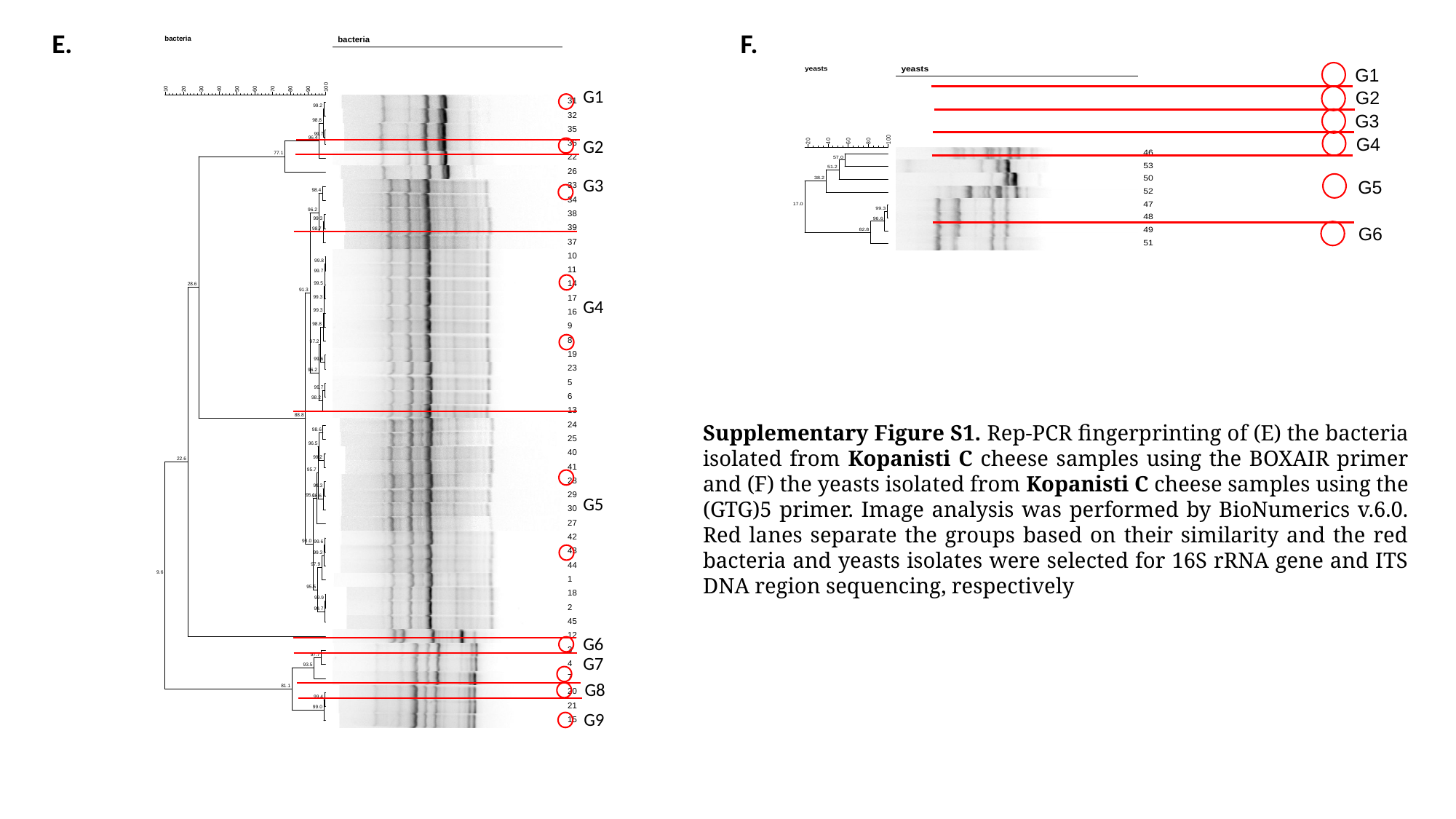

G1
G2
G3
G4
G5
G6
F.
E.
G1
G2
G3
G4
G5
G6
G7
G8
G9
Supplementary Figure S1. Rep-PCR fingerprinting of (E) the bacteria isolated from Kopanisti C cheese samples using the BOXAIR primer and (F) the yeasts isolated from Kopanisti C cheese samples using the (GTG)5 primer. Image analysis was performed by BioNumerics v.6.0. Red lanes separate the groups based on their similarity and the red bacteria and yeasts isolates were selected for 16S rRNA gene and ITS DNA region sequencing, respectively

## Slide 4
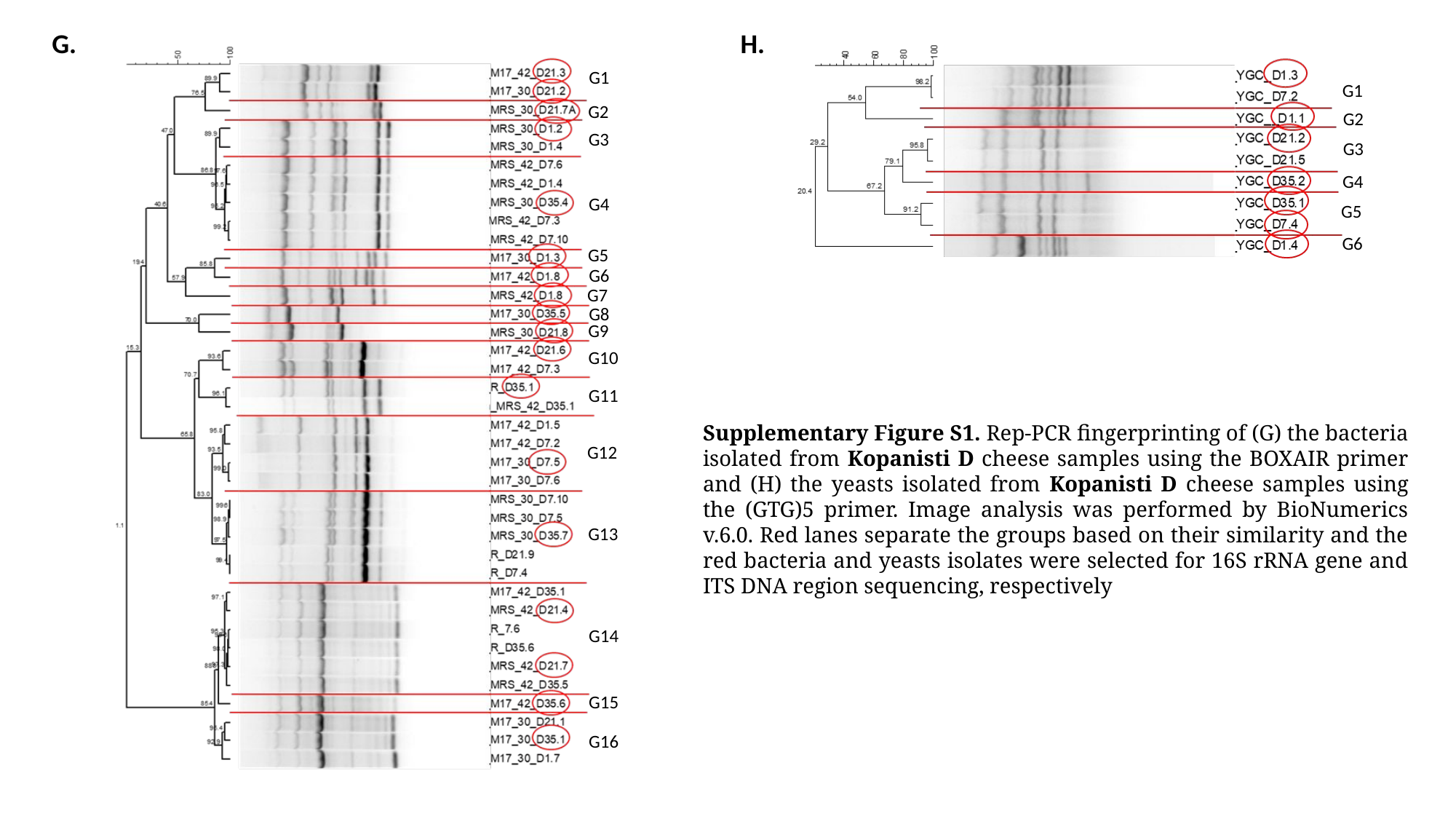

H.
G.
G1
G2
G3
G4
G5
G6
G7
G8
G9
G10
G11
G12
G13
G14
G15
G16
G1
G2
G3
G4
G5
G6
Supplementary Figure S1. Rep-PCR fingerprinting of (G) the bacteria isolated from Kopanisti D cheese samples using the BOXAIR primer and (H) the yeasts isolated from Kopanisti D cheese samples using the (GTG)5 primer. Image analysis was performed by BioNumerics v.6.0. Red lanes separate the groups based on their similarity and the red bacteria and yeasts isolates were selected for 16S rRNA gene and ITS DNA region sequencing, respectively
