# Supplementary material for: The Impact of Different Inoculation Schemes on the Microbiota, Physicochemical and Sensory Characteristics of Greek Kopanisti Cheese throughout Production and Ripening
Source: Microorganisms. 2022 Dec 26;11(1):66. doi: 10.3390/microorganisms11010066 (PMC9863000; doi:10.3390/microorganisms11010066)
Supplement: Supplementary file 1 [file microorganisms-11-00066-s001.zip › Supplementary Tables S1 and S5.pdf]

**Supplementary Table S1**. Microbiological evaluation (mean log CFU mL$^{-1}$ or g$^{-1}$ ± SD) of pasteurized milk, Mana Kopanisti and Kopanisti cheese samples during ripening

| Sample | Day | Total mesophilic bacteria | Mesophilic lactobacilli | Thermophilic lactobacilli | Mesophilic cocci | Thermophilic cocci | NSLAB | Enterococci | Psychrotrophic bacteria | Enterobacteria | Yeasts |
|---|---|---|---|---|---|---|---|---|---|---|---|
| Pasteurized milk | - | 3.7 ± 0.7 | 2.5 ± 1.1 | 2.7 ± 0.9 | 3.5 ± 0.6 | 3.3 ± 0.7 | BDL | BDL | 0.8 ± 1.7 | BDL | BDL |
| Mana Kopanisti | - | BDL | BDL | BDL | BDL | BDL | BDL | BDL | BDL | BDL | BDL |
| Kopanisti A | 1 | 9.5 ± 0.1$^{Aa}$ | 9.6 ± 0.2$^{Aa}$ | 9.6 ± 0.1$^{Aa}$ | 9.6 ± 0.2$^{Aa}$ | 9.6 ± 0.1$^{Aa}$ | 8.2 ± 0.3$^{Aa}$ | 0.9 ± 1.6$^{Aa}$ | 4.6 ± 0.4$^{ABa}$ | 2.3 ± 0.4$^{Aa}$ | 3.8 ± 0.3$^{Aa}$ |
| | 7 | 9.0 ± 0.3$^{ABb}$ | 8.7 ± 0.1$^{Ab}$ | 8.8 ± 0.2$^{Ab}$ | 8.9 ± 0.3$^{Ab}$ | 8.8 ± 0.2$^{Ab}$ | 8.6 ± 1.0$^{Aab}$ | BDL | 5.9 ± 0.3$^{Aac}$ | 0.7 ± 1.2$^{Aa}$ | 7.4 ± 0.5$^{Bbc}$ |
| | 21 | 9.2 ± 0.1$^{Aab}$ | 9.1 ± 0.1$^{Ac}$ | 9.0 ± 0.2$^{Aab}$ | 9.1 ± 0.0$^{Aab}$ | 8.9 ± 0.2$^{Ab}$ | 9.1 ± 0.2$^{Ab}$ | BDL | 7.5 ± 0.4$^{Ab}$ | 1.6 ± 2.8$^{Aa}$ | 7.9 ± 0.4$^{Ab}$ |
| | 35 | 8.7 ± 0.5$^{ABb}$ | 8.7 ± 0.4$^{ABb}$ | 8.4 ± 0.7$^{Ab}$ | 8.8 ± 0.4$^{ABb}$ | 8.4 ± 0.5$^{ABb}$ | 8.7 ± 0.4$^{Ab}$ | BDL | 6.3 ± 0.9$^{Abc}$ | BDL | 6.6 ± 1.0$^{ABc}$ |
| Kopanisti B | 1 | 9.6 ± 0.2$^{Aa}$ | 9.4 ± 0.2$^{Aa}$ | 9.4 ± 0.2$^{Aa}$ | 9.5 ± 0.2$^{Aa}$ | 9.5 ± 0.1$^{Aa}$ | 8.2 ± 0.2$^{Aa}$ | BDL | 4.0 ± 0.0$^{Aa}$ | 0.7 ± 1.3$^{Aa}$ | 3.8 ± 0.3$^{Aa}$ |
| | 7 | 8.6 ± 0.2$^{Ab}$ | 8.5 ± 0.2$^{Ab}$ | 8.5 ± 0.2$^{Ab}$ | 8.6 ± 0.2$^{Ab}$ | 8.6 ± 0.3$^{Ab}$ | 8.6 ± 0.3$^{Aab}$ | BDL | 4.7 ± 0.6$^{Aa}$ | BDL | 5.0 ± 0.6$^{Ab}$ |
| | 21 | 9.0 ± 0.1$^{Ac}$ | 9.0 ± 0.3$^{Aab}$ | 9.0 ± 0.2$^{Aab}$ | 9.0 ± 0.2$^{Aab}$ | 8.8 ± 0.4$^{Aab}$ | 9.0 ± 0.2$^{Ab}$ | 1.3 ± 2.3$^{Aa}$ | 7.5 ± 0.4$^{Ab}$ | 0.5 ± 0.9$^{Aa}$ | 7.8 ± 0.4$^{Ac}$ |
| | 35 | 9.3 ± 0.3$^{Aac}$ | 9.0 ± 0.3$^{Aab}$ | 8.9 ± 0.4$^{Aab}$ | 9.1 ± 0.4$^{Aab}$ | 8.9 ± 0.5$^{Aab}$ | 9.0 ± 0.3$^{Ab}$ | BDL | 7.7 ± 1.4$^{Ab}$ | BDL | 7.9 ± 1.3$^{Ac}$ |
| Kopanisti C | 1 | 9.6 ± 0.2$^{Aa}$ | 9.5 ± 0.2$^{Aa}$ | 9.2 ± 0.8$^{Aa}$ | 9.5 ± 0.2$^{Aa}$ | 9.7 ± 0.2$^{Aa}$ | 7.1 ± 0.4$^{Ca}$ | 0.8 ± 1.4$^{Aa}$ | 3.5 ± 0.4$^{Aa}$ | 1.5 ± 0.9$^{Aa}$ | 3.3 ± 0.2$^{Aa}$ |
| | 7 | 8.9 ± 0.2$^{ABb}$ | 8.3 ± 0.1$^{Ab}$ | 8.3 ± 0.2$^{ACb}$ | 8.5 ± 0.2$^{Ab}$ | 8.4 ± 0.3$^{Ab}$ | 8.3 ± 0.2$^{ABb}$ | BDL | 7.2 ± 0.7$^{Cb}$ | 0.3 ± 0.6$^{Aab}$ | 7.5 ± 0.2$^{Bb}$ |
| | 21 | 9.1 ± 0.1$^{Aab}$ | 9.1 ± 0.1$^{Ac}$ | 9.0 ± 0.1$^{Aab}$ | 9.0 ± 0.0$^{Aab}$ | 9.0 ± 0.1$^{Ac}$ | 9.1 ± 0.1$^{Ac}$ | BDL | 7.2 ± 0.6$^{Ab}$ | BDL | 7.3 ± 0.4$^{Abc}$ |
| | 35 | 8.7 ± 0.4$^{ABb}$ | 8.7 ± 0.3$^{ABd}$ | 8.5 ± 0.5$^{Aab}$ | 8.7 ± 0.3$^{ABb}$ | 8.6 ± 0.4$^{ABbc}$ | 8.5 ± 0.3$^{ABb}$ | BDL | 6.6 ± 1.2$^{Ab}$ | BDL | 6.3 ± 1.2$^{ABc}$ |
| Kopanisti D | 1 | 9.4 ± 0.1$^{Aa}$ | 7.9 ± 0.2$^{Ba}$ | 7.8 ± 0.2$^{Ba}$ | 9.2 ± 0.5$^{Aa}$ | 9.5 ± 0.1$^{Aa}$ | BDL | 2.1 ± 2.4$^{Aa}$ | 6.3 ± 1.8$^{Ba}$ | 1.7 ± 1.4$^{Aa}$ | 3.4 ± 0.3$^{Aa}$ |
| | 7 | 9.2 ± 0.0$^{Ba}$ | 6.9 ± 0.5$^{Bb}$ | 7.6 ± 0.1$^{Ba}$ | 8.9 ± 1.0$^{Aa}$ | 9.4 ± 0.2$^{Ba}$ | 6.9 ± 1.7$^{Bb}$ | BDL | 8.5 ± 0.0$^{Ba}$ | BDL | 7.3 ± 0.7$^{Bb}$ |
| | 21 | 8.7 ± 0.0$^{Bb}$ | 8.7 ± 0.1$^{ABc}$ | 8.0 ± 0.3$^{Ba}$ | 8.9 ± 0.2$^{Aa}$ | 8.7 ± 0.1$^{Ab}$ | 8.7 ± 0.1$^{Bc}$ | BDL | 7.7 ± 0.3$^{Aa}$ | BDL | 6.1 ± 0.6$^{Bb}$ |
| | 35 | 8.3 ± 0.0$^{Bb}$ | 8.3 ± 0.3$^{Bac}$ | 7.2 ± 0.1$^{Ba}$ | 8.3 ± 0.3$^{Ba}$ | 8.1 ± 0.2$^{Bc}$ | 8.2 ± 0.2$^{Bbc}$ | BDL | 6.7 ± 1.3$^{Aa}$ | BDL | 5.0 ±1.5$^{Bab}$ |

Different capital letters within the same column indicate statistically significant differences ($P < 0.05$) among the Kopanisti samples (A-D) at the same day of ripening (1-35)

Different lowercase letters within the same column indicate statistically significant differences ($P < 0.05$) among the days of ripening (1-35) per Kopanisti sample (A-D)

Below Detection Limit (BDL):

DL for pasteurized milk was < 1.0 log CFU mL$^{-1}$ for total mesophilic bacteria, mesophilic and thermophilic lactobacilli and cocci, NSLAB, psychrotrophic bacteria and enterobacteria (pouring inoculation method) and 1.0 log CFU mL$^{-1}$ for enterococci and yeasts (spreading inoculation method)

DL for Mana Kopanisti and Kopanisti was 1.0 log CFU g$^{-1}$ for total mesophilic bacteria, mesophilic and thermophilic lactobacilli and cocci, NSLAB, psychrotrophic bacteria and enterobacteria (pouring inoculation method) and 2.0 log CFU g$^{-1}$ for enterococci and yeasts (spreading inoculation method)

**Supplementary Table S5**. Physicochemical evaluation (mean values ± SD) of Mana Kopanisti and Kopanisti cheese samples during ripening

| Kopanisti | Day | pH | Moisture (% w/w) | SIM (% w/w) | Ash (% w/w) | FDM (% w/w) | Protein (% w/w) |
|---|---|---|---|---|---|---|---|
| Mana Kopanisti | - | 5.16 ± 0.03 | 42.39 ± 0.75 | 10.12 ± 0.20 | 6.15 ± 0.06 | 53.44 ± 0.90 | 22.52 ± 1.32 |
| A | 1 | $4.16 \pm 0.03^{Aa}$ | $58.07 \pm 0.80^{Aa}$ | $3.28 \pm 0.11^{Aa}$ | $2.91 \pm 0.10^{Aa}$ | $48.47 \pm 1.66^{Aa}$ | $15.61 \pm 1.45^{Aa}$ |
| | 7 | $4.38 \pm 0.13^{Aa}$ | $58.45 \pm 2.84^{Aa}$ | $3.39 \pm 0.20^{Aa}$ | $2.95 \pm 0.12^{Aa}$ | $51.14 \pm 2.87^{Aa}$ | $15.88 \pm 1.31^{Aa}$ |
| | 21 | $5.26 \pm 0.15^{Ab}$ | $56.90 \pm 2.65^{Aa}$ | $3.53 \pm 0.18^{Aa}$ | $3.01 \pm 0.10^{Aa}$ | $52.62 \pm 1.46^{Aa}$ | $16.64 \pm 1.24^{Aa}$ |
| | 35 | $5.29 \pm 0.25^{Ab}$ | $56.31 \pm 1.45^{Aa}$ | $3.62 \pm 0.31^{Aa}$ | $3.21 \pm 0.11^{Aa}$ | $52.89 \pm 2.36^{Aa}$ | $17.04 \pm 0.85^{Aa}$ |
| B | 1 | $4.61 \pm 0.15^{Ba}$ | $55.23 \pm 1.23^{Aa}$ | $3.80 \pm 0.50^{Aa}$ | $3.21 \pm 0.47^{Aa}$ | $47.95 \pm 2.84^{Aa}$ | $15.65 \pm 1.34^{Aa}$ |
| | 7 | $4.45 \pm 0.11^{Aa}$ | $53.48 \pm 1.44^{Aab}$ | $4.18 \pm 0.46^{Aa}$ | $3.25 \pm 0.46^{Aa}$ | $50.87 \pm 1.22^{Aa}$ | $16.73 \pm 1.52^{Aa}$ |
| | 21 | $4.69 \pm 0.52^{Aa}$ | $49.60 \pm 1.73^{Abc}$ | $5.01 \pm 0.62^{Bab}$ | $3.40 \pm 0.54^{Aa}$ | $51.24 \pm 0.78^{Aa}$ | $18.72 \pm 1.24^{Aab}$ |
| | 35 | $5.31 \pm 0.73^{Aa}$ | $45.23 \pm 3.25^{Bc}$ | $6.11 \pm 0.98^{Bb}$ | $3.48 \pm 0.46^{Aa}$ | $51.73 \pm 0.98^{Aa}$ | $20.95 \pm 1.34^{Bb}$ |
| C | 1 | $4.18 \pm 0.02^{Aa}$ | $58.39 \pm 3.73^{Aa}$ | $3.35 \pm 0.32^{Aa}$ | $2.76 \pm 0.19^{Aa}$ | $52.22 \pm 2.39^{Aa}$ | $15.94 \pm 0.85^{Aa}$ |
| | 7 | $4.21 \pm 0.04^{Aa}$ | $57.92 \pm 5.70^{Aa}$ | $3.40 \pm 0.17^{Aa}$ | $2.82 \pm 0.06^{Aa}$ | $53.24 \pm 1.54^{Aa}$ | $16.14 \pm 0.74^{Aa}$ |
| | 21 | $4.85 \pm 0.32^{Aa}$ | $57.26 \pm 2.52^{Aa}$ | $3.57 \pm 0.19^{Aa}$ | $2.89 \pm 0.09^{Aa}$ | $54.54 \pm 1.68^{Aa}$ | $16.48 \pm 0.35^{Aa}$ |
| | 35 | $4.88 \pm 0.42^{Aa}$ | $56.67 \pm 2.86^{Aa}$ | $3.70 \pm 0.41^{Aa}$ | $3.05 \pm 0.11^{Aa}$ | $55.23 \pm 1.99^{Aa}$ | $16.98 \pm 0.62^{Aa}$ |
| D | 1 | $4.69 \pm 0.19^{Ba}$ | $57.48 \pm 2.19^{Aa}$ | $2.95 \pm 0.70^{Aa}$ | $2.68 \pm 0.54^{Aa}$ | $50.30 \pm 2.85^{Aa}$ | $15.89 \pm 0.62^{Aa}$ |
| | 7 | $4.74 \pm 0.17^{Aa}$ | $56.67 \pm 2.20^{Aa}$ | $3.07 \pm 0.69^{Aa}$ | $2.75 \pm 0.54^{Aa}$ | $51.73 \pm 2.43^{Aa}$ | $16.05 \pm 0.35^{Aa}$ |
| | 21 | $4.49 \pm 0.26^{Aa}$ | $54.99 \pm 2.47^{Aa}$ | $3.38 \pm 0.87^{Aa}$ | $2.88 \pm 0.36^{Aa}$ | $53.00 \pm 0.88^{Aa}$ | $17.21 \pm 0.07^{Aa}$ |
| | 35 | $4.47 \pm 0.30^{Aa}$ | $53.05 \pm 1.67^{Aa}$ | $3.78 \pm 0.87^{Aa}$ | $3.11 \pm 0.54^{Aa}$ | $53.45 \pm 2.23^{Aa}$ | $17.79 \pm 0.18^{Aa}$ |

Salt in moisture (SIM)
Fat in dry matter (FDM)
Different capital letters within the same column indicate statistically significant differences ($P < 0.05$) among the Kopanisti samples (A-D) at the same day of ripening (1-35).
Different lowercase letters within the same column indicate statistically significant differences ($P < 0.05$) among the days of ripening (1-35) per Kopanisti sample (A-D).
